# Polycystic Ovary Syndrome: An Evolutionary Adaptation to Lifestyle and the Environment

**DOI:** 10.3390/ijerph19031336

**Published:** 2022-01-25

**Authors:** Jim Parker, Claire O’Brien, Jason Hawrelak, Felice L. Gersh

**Affiliations:** 1 School of Medicine, University of Wollongong, Wollongong 2500, Australia; 2 Faculty of Science and Technology, University of Canberra, Bruce 2617, Australia; Claire.obrien@canberra.edu.au; 3 College of Health and Medicine, University of Tasmania, Hobart 7005, Australia; Jason.Hawrelak@utas.edu.au; 4 College of Medicine, University of Arizona, Tucson, AZ 85004, USA; felicelgersh@yahoo.com

**Keywords:** polycystic ovary syndrome, evolution, insulin resistance, infertility, toxins, endocrine-disrupting chemicals, environment, lifestyle, diet

## Abstract

Polycystic ovary syndrome (PCOS) is increasingly recognized as a complex metabolic disorder that manifests in genetically susceptible women following a range of negative exposures to nutritional and environmental factors related to contemporary lifestyle. The hypothesis that PCOS phenotypes are derived from a mismatch between ancient genetic survival mechanisms and modern lifestyle practices is supported by a diversity of research findings. The proposed evolutionary model of the pathogenesis of PCOS incorporates evidence related to evolutionary theory, genetic studies, in utero developmental epigenetic programming, transgenerational inheritance, metabolic features including insulin resistance, obesity and the apparent paradox of lean phenotypes, reproductive effects and subfertility, the impact of the microbiome and dysbiosis, endocrine-disrupting chemical exposure, and the influence of lifestyle factors such as poor-quality diet and physical inactivity. Based on these premises, the diverse lines of research are synthesized into a composite evolutionary model of the pathogenesis of PCOS. It is hoped that this model will assist clinicians and patients to understand the importance of lifestyle interventions in the prevention and management of PCOS and provide a conceptual framework for future research. It is appreciated that this theory represents a synthesis of the current evidence and that it is expected to evolve and change over time.

## 1. Introduction

Polycystic ovary syndrome is a reversible metabolic condition that makes a significant contribution to the global epidemic of lifestyle-related chronic disease [1,2,3]. Many of these chronic diseases share a similar pathogenesis involving the interaction of genetic and environmental factors [4,5,6]. The revised International Guidelines for the assessment and management of women with PCOS emphasize that the associated metabolic dysfunction and symptoms should initially be addressed via lifestyle interventions [7]. A unified evolutionary model proposes that PCOS represents a mismatch between our ancient biology and modern lifestyle.

Evolutionary medicine is an emerging discipline involving the study of evolutionary processes that relate to human traits and diseases and the incorporation of these findings into the practice of medicine [8]. Evolutionary medicine brings together interdisciplinary research to inform clinical medicine based on the influence of evolutionary history on human health and disease [9]. Previous utilization of the principles of evolutionary medicine has been limited to monogenetic diseases (cystic fibrosis, sickle cell anemia, phenylketonuria and many others), drug resistance of microorganisms, tumor growth and chemoresistance [8]. Future insights into the application of evolutionary research offers the potential to improve and personalize the established medical and scientific approaches to complex chronic diseases such as type 2 diabetes, metabolic syndrome and PCOS [5,9].

The evolutionary origins of complex chronic diseases incorporate considerations of relative reproductive fitness, mismatch between our biological past and modern environment, trade-offs involving combinations of genetic traits, and evolutionary conflicts [8,10]. These evolutionary factors are relevant when analyzing the contributors to the pathogenesis of PCOS in modern and modernizing societies that result in a mismatch between our rapid cultural evolution with our slow biological evolution [11,12]. The unique cultural evolution of humans does not have a plausible analogue in most other species and is increasingly recognized to play a significant role in the pathogenesis of metabolic diseases such as PCOS [5,13,14,15,16,17].

Polycystic ovary syndrome is a complex multisystem condition with metabolic, endocrine, psychological, fertility and pregnancy-related implications at all stages of life [7,18]. The majority of women with PCOS manifest multiple metabolic features including obesity, insulin resistance (IR), hyperlipidemia and hyperandrogenism [19,20]. PCOS results in an increased risk of developing metabolic disease (type 2 diabetes, non-alcoholic fatty liver disease [NAFLD] and metabolic syndrome), cardiovascular disease, cancer, a wide array of pregnancy complications (deep venous thrombosis, pre-eclampsia, gestational diabetes [GDM], macrosomia, growth restriction, miscarriage, stillbirth and preterm labor) and psychological problems (anxiety, depression) [6,21,22,23,24,25]. PCOS is part of a cluster of inter-related metabolic conditions and makes a significant contribution to the chronic disease epidemic.

Extensive research suggests that the etiology of PCOS involves an interaction between environmental factors and gene variants, although it has been suggested that genetic factors contribute less than 10% to disease susceptibility [26,27,28]. A large number of genetic and genome-wide association studies (GWAS) have identified common gene loci associated with PCOS phenotypes in different ethnic populations [29,30,31]. These appear to be normal gene variants or polymorphisms, given the frequency and type of genes that have been identified. PCOS is therefore viewed as a polygenic trait that results from an interaction between susceptible genomic variants and the environment.

PCOS effects upward of 10% of reproductive-aged women, estimated at over 200 million women worldwide [32,33]. PCOS is thought to be increasing in incidence in both developing and developed nations as a result of lifestyle-related changes in diet quality, reduced physical activity, ubiquitous environmental endocrine-disrupting chemicals (EDC), altered light exposures, sleep disturbance, heightened levels of stress and other environmental factors [11,34,35,36,37,38]. These factors, and the high prevalence of PCOS, suggest that there could be an evolutionary basis for the syndrome [15,16,39]. Evolutionary medicine has changed the paradigm for understanding PCOS, acknowledging many of the contributing lifestyle and environmental factors that facilitate the observed metabolic and clinical features and that are also shared with related metabolic diseases [8]. These “mismatch disorders” are estimated to make a significant contribution to chronic disease in developed countries and a growing proportion of disability and death in developing nations [3]. According to the Global Burden of Disease Study, the human diet is now the leading risk factor for morbidity and mortality worldwide [3]. In keeping with these findings, diet is recognized as one of the major contributors to the growing prevalence of PCOS globally [7,40].

Dietary and environmental factors are hypothesized to have an impact on developmental programming of susceptible gene variants in women with PCOS [41,42,43]. Extensive experimental evidence suggests that prenatal androgen exposure may play a role in the pathogenesis of PCOS-like syndromes in animal models [19,44,45,46]. The discovery of naturally occurring PCOS phenotypes in non-human primates supports a survival advantage of a hyperandrogenic, insulin resistant phenotype with delayed fertility [47]. In humans, the origin of excess androgens may be from maternal, fetal or placental sources. In addition, emerging and concerning evidence suggests that EDC may contribute to altered fetal programming and play a role in the pathogenesis of PCOS [41,48].

In utero genomic programming of metabolic and endocrine pathways can increase the susceptibility of offspring to develop PCOS following exposure to specific nutritional and environmental conditions [45]. This view of the pathogenesis of PCOS is consistent with the Developmental Origins of Health and Disease (DOHaD) model proposed by Neel [49]. Postnatal exposure to lifestyle and environmental factors, such as poor-quality diet and EDC, may activate epigenetically programmed pathways that further promote the observed features of PCOS. Dietary and lifestyle interventions have demonstrated that many of the clinical, metabolic and endocrine features of PCOS can be reversed [7,50,51].

Lifestyle-induced changes in the gastrointestinal tract microbiome are another significant factor in the etiology of PCOS [52,53]. Dysbiosis of the gut microbiota has been hypothesized to play a role in increased gastrointestinal permeability, initiating chronic inflammation, insulin resistance (IR) and hyperandrogenism [40]. Numerous studies have reported reduced alpha diversity of the microbiome that has been associated with the metabolic, endocrine and clinical features observed in women with PCOS [54,55]. The resulting dysbiosis has been shown to be reversible after interventions aimed at improving diet quality or treatment with probiotics or synbiotics [50,51,56,57,58].

**Figure 1:**
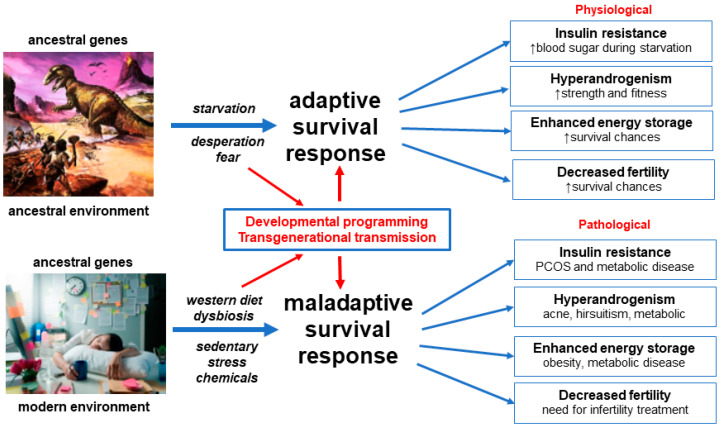
Evolutionary model of the pathogenesis of polycystic ovary syndrome. Adapted with permission from Ref. [12]. 2021 Journal of ACNEM.

A unified evolutionary theory of the pathogenesis of PCOS proposes that ancient genetic polymorphisms that were aligned with the environment of that era, resulted in an adaptive survival advantage in offspring in ancestral populations [14,15,16,28]. When these same genetic variants are exposed to modern lifestyle and environmental influences, maladaptive physiological responses occur. The prior advantages of insulin resistance, hyperandrogenism, enhanced energy storage and reduced fertility in ancestral populations become pathological and result in the observed features of PCOS in contemporary women (Figure 1).

## 2. Materials and Methods

The literature search focused on research publications related to the pathogenesis of PCOS using the keywords listed above and related mesh terms for data on the evolutionary aspects of PCOS, genetic studies, in utero developmental epigenetic programming, transgenerational inheritance, metabolic features including insulin resistance, obese and lean PCOS phenotypes, reproductive changes and subfertility, impact of the microbiome and dysbiosis, possible effects of endocrine-disrupting chemical exposure and the influence of lifestyle factors such as diet and physical activity. The databases searched included PubMed, Scopus, Cochrane and Google Scholar. Relevant papers were selected, and citation searches were performed.

The present manuscript synthesizes the findings into a unified evolutionary model. The following text is presented as a narrative review of factors involved in the pathogenesis of PCOS and is discussed in ten main subject areas that provide the rationale for the development of a unified model. 1. Evolution 2. Genetics 3. Developmental Epigenetic Programming 4. Microbiome and Dysbiosis 5. Insulin resistance 6. Obesity and the lean paradox 7. Endocrine-Disrupting Chemical Exposure 8. Lifestyle contributors to the pathogenesis of PCOS 9. Circadian Rhythm Disruption and PCOS 10. Conceptual Framework and Summary of the Unified Evolutionary Model.

## 3. Pathogenesis of PCOS

### 3.1. Evolution

The description of PCOS phenotypes can be found in medical records from antiquity and the modern syndrome was described over 80 years ago [17,59]. Nevertheless, there is ongoing debate regarding the evolutionary origins of PCOS [15,16,17,39,60,61,62,63,64]. PCOS susceptibility alleles may have arisen in our phylogenetic ancestors, in the hunter–gatherer Paleolithic period of the Stone Age, after the Neolithic Agricultural Revolution or following the Industrial Revolution [16,17]. From an evolutionary perspective, nearly all genetic variants that influence disease risk have human-specific origins, but the systems they relate to have ancient roots in our evolutionary ancestors [8]. Regardless of the precise timing of the origin of PCOS in humans, the complex metabolic and reproductive gene variants identified in women with PCOS relate to ancient evolutionary-conserved metabolic and reproductive survival pathways [15,29]. Although evolutionary hypotheses about disease vulnerability are impossible to prove they have the potential to frame medical thinking and direct scientific research for the proximate causes of disease [15,60].

Multiple hypotheses have been proposed regarding the evolutionary origins of PCOS and related metabolic diseases [8,60,63]. These hypotheses are focused on the relative importance of metabolic survival adaptations versus improved reproductive success, or a combination of both. A detailed analysis of these hypotheses, and the complexities of the evolutionary considerations, have been reviewed elsewhere and is beyond the scope of the present review [8,60]. One common theme is that PCOS may be viewed as a “conditional phenotype” where a specific set of conditions has unmasked normally unexpressed or partly expressed genetic pathways, which then provide a survival advantage under certain environmental conditions [14,16].

All organisms have physiological adaptive responses to deal with changing environmental conditions (starvation, fasting, physical threat, stress and infection) and the varying demands of internal physiological states (pregnancy, lactation and adolescence) [14,65]. It has been proposed that the PCOS phenotype may have been invoked in specific environmental conditions in ancestral populations as a short, medium or even long-term adaptive survival mechanism [15,16,17]. The view of PCOS as a conditional phenotype proposes that these physiological responses become pathological in our modern environment due to factors such as food abundance, reduced physical activity, circadian disruption, stress and environmental chemical exposure. The transgenerational evolutionary theory of the pathogenesis of PCOS encompasses all of the above ideas to explain the observed pathophysiological and clinical features of PCOS [28].

It is generally accepted that almost all pre-industrial societies and animal populations experienced seasonal or unpredictable episodes of food shortage that applied evolutionary pressure to develop metabolic and reproductive adaptive survival responses [17,49]. It is also appreciated that metabolic and reproductive pathways are interconnected and involve reciprocal feedback control mechanisms [66,67,68]. During periods of starvation, anorexia or excessive weight gain, reproduction is down-regulated and ovulation becomes irregular or ceases [69,70]. Similarly, metabolic function is coordinated with the menstrual cycle to ensure optimal physiological conditions for fertilization, implantation, pregnancy, parturition and lactation [71]. Recent research has elaborated on the details of how some of these complex regulatory mechanisms interact using specific hormonal, nutrient sensing and intracellular signaling networks [72,73,74].

Details of the mechanisms underlying the proposed adaptive survival advantages of IR, hyperandrogenism, enhanced energy storage and subfertility have been obtained from paleolithic records, animal models and human populations exposed to adverse environmental conditions such as war and famine-inflicted starvation [14,16,62,63]. Multiple lines of evidence support the maladaptive response of human populations to rapidly changing nutritional, physical, psychological and cultural environments, in the modern world [5,11,14,75]. These “adaptations” result in pathological responses to IR, hyperandrogenism, enhanced energy storage and ovulation (Figure 1).

Theories of evolutionary mismatch have also been advanced to explain all of the cluster of metabolic diseases associated with PCOS (type 2 diabetes, metabolic syndrome, NAFLD and cardiovascular disease) and follow the same set of basic principles and explanations [14,76]. This common body of evolutionary evidence is supported by the increasing incidence of metabolic-related disease, such as diabetes and obesity, in developed countries and in developing nations adopting a Western diet and lifestyle [11,77]. In addition, the demonstrated reversibility of PCOS and related metabolic and biochemical features following changes in diet, increased physical activity and other lifestyle interventions, adds further support to a transgenerational evolutionary model [50,51].

### 3.2. Genetics

The heritable nature of PCOS has been proposed since the 1960′s following a range of familial, twin and chromosomal studies [78,79,80]. Cytogenetic studies failed to identify karyotypic abnormalities and genetic studies did not show a monogenic inheritance pattern following examination of candidate genes [81,82]. In addition, two or more phenotypes can be present in the same family suggesting that some of the phenotypic differences could be accounted for by variable expression of the same shared genes [81,83].

The mapping of the human genome in 2003 [84] and the publication of the human haplotype map (more than one million single nucleotide polymorphisms of common genetic variants) in 2005 [85], lead to the realization that most DNA variation is shared by all humans and is inherited as blocks of linked genes (linkage disequilibrium) [86]. These advances enabled a revolution in case-control studies and the development of GWAS which map the entire human genome looking for susceptibility genes for complex traits such as obesity, type 2 diabetes and PCOS [81].

The first PCOS GWAS was published in 2010 and demonstrated 11 gene loci associated with PCOS [87]. Additional loci have subsequently been found in several different ethnic groups [86,88]. The first GWAS analysis of quantitative traits was published in 2015 and showed that a variant (rs11031006) was associated with luteinizing hormone levels [88]. The largest GWAS included a meta-analysis of 10,074 PCOS cases and 103,164 controls and identified 19 loci that confer risk for PCOS [29]. The genes associated with these loci involve gonadotrophin action, ovarian steroidogenesis, insulin resistance and type 2 diabetes susceptibility genes. The first GWAS using electronic health record-linked biobanks has introduced greater investigative power and identified 2 additional loci [89]. These variants were associated with polycystic ovaries and hyperandrogenism (rs17186366 near *SOD2*) and oligomenorrhoea and infertility (rs144248326 near *WWTR1*) [89]. In addition to identifying common gene variants for PCOS phenotypes, finding the same signals (THADA, YAP1 and c9orf3) in Chinese and European populations suggests that PCOS is an ancient trait that was present before humans migrated out of Africa [81].

More recently Mendelian randomization (MR) studies have been used to explore the potential causative association between gene variants identified in GWAS and PCOS [90,91]. Many of the gene variants identified in GWAS are located in non-coding regions of DNA [92]. The genes or functional DNA elements through which these variants exert their effects are often unknown. Mendelian randomization is a statistical methodology used to jointly analyze GWAS and quantitative gene loci to test for association between gene expression and a trait, due to a shared or potentially causal variant at a specific locus [93]. A detailed analysis of MR methodology and the limitations of this statistical tool is beyond the scope of the present review. Although MR studies have the potential to infer causation it is recognized that they also have limitations in PCOS research [90]. Nevertheless, preliminary evidence suggests that several genes related to obesity, metabolic and reproductive function, may play a causal role in the pathogenesis of PCOS [90,91].

Decades of genetic research has therefore characterized PCOS as a polygenic trait that results from interactions between the environment and susceptible genomic traits [27,29,79,88]. The failure to identify a qualitative or monogenic inheritance pattern and the findings from GWAS, MR, familial and twin studies, suggests that the heritability of PCOS is likely to be due to the combination of multiple genes with small effect size, as has been found with obesity and type 2 diabetes [79,80,94,95,96]. Polygenic traits are the result of gene variants that represent one end of the bell-shaped normal distribution curve of continuous variation in a population [97]. From an evolutionary perspective, women with PCOS may represent the “metabolic elite” end of the normal distribution curve, being able to efficiently store energy in periods of food abundance and down-regulate fertility in times of food scarcity, or even in anticipation of reduced seasonal food availability as a predictive adaptive response [16,17,60].

The realization that PCOS is a quantitative trait (phenotype determined by multiple genes and environmental factors) has far-reaching implications for the diagnosis, treatment and prevention of symptoms and pathology associated with PCOS. The implications require a shift in thinking about PCOS as a “disease” to a variation of normal metabolic and reproductive function. This shift invites a change in vocabulary from talking about “disorder” and “risk” to talking about “expression” and “variability” [97]. This new understanding supports and reinforces an evolutionary model of the pathogenesis of PCOS. In keeping with this model, multiple lines of evidence suggest that inherited PCOS gene variants are developmentally programmed in a way that primes them for activation by nutritional and environmental factors in postnatal life [41,42,98].

### 3.3. Developmental Epigenetic Programming

The developmental programming of PCOS represents changes in gene expression that occur during critical periods of fetal development [99]. Following fertilization, most parental epigenetic programming is erased and dramatic epigenomic reprogramming occurs [100]. This results in transformation of the parental epigenome to the zygote epigenome and determines personalized gene function. Compelling evidence shows that a wide range of maternal, nutritional and environmental factors can effect fetal development during these critical periods of programming [44,98,99,101,102]. These include hormones, vitamins, diet-derived metabolites and environmental chemicals [48,98,103,104]. In addition, epigenetic reprogramming of germ-line cells can lead to transgenerational inheritance resulting in phenotypic variation or pathology in the absence of continued direct exposure [98].

Experimental studies in primates, sheep, rats and mice show that PCOS-like syndromes can be induced by a range of treatments including androgens, anti-Mullerian hormone and letrozole [19,44,46]. Nevertheless, there is significant debate regarding when an animal model qualifies as PCOS-like [105]. The model used and the method of induction of PCOS phenotypes therefore needs to be carefully scrutinized when generalizing findings from animal research to women with PCOS. Most of the animal and human research on the developmental origins of PCOS has focused on the role of prenatal androgen exposure. This has been extensively reviewed in numerous previous publications [41,46]. This research has resulted in a proposed “two hit” hypothesis for the development of PCOS phenotypes [43,45]. The “first hit” involves developmental programming of inherited susceptibility genes and the “second hit” arises due to lifestyle and environmental influences in childhood, adolescence and adulthood [41,106].

If PCOS is a quantitative trait involving normal gene variants, as suggested by the evolutionary considerations and findings from genetic research, then the “first hit” may result from normal developmental programming events as occurs with other gene variants [102]. According to this hypothesis, the polygenic susceptibility genes would be normally “activated” and “primed” to respond to future maternal and environmental conditions and exposures, as would be the case with many other normal genes [28]. In addition, the susceptibility alleles may be “activated” or “functionally enhanced” by a range of maternal and environmental factors, as is usually presumed to be the case in PCOS [5,14,102]. This developmental plasticity would provide a mechanism for a predictive adaptive response, based on inputs from the maternal environment that could be used to program metabolic and reproductive survival pathways, to better prepare the offspring for the future world in which they may be expected to live [107].

Parental lifestyle factors including diet, obesity, smoking and endocrine-disrupting chemicals, have all been shown to modulate disease risk later in life [104,108,109]. The original description of the fetal origin’s hypothesis proposed that poor maternal nutrition would increase fetal susceptibility to the effects of a Western-style diet later in life [49]. Subsequent studies have confirmed that maternal exposure to either nutrient excess or deficit, can have long-term consequences for the health of the progeny [104]. Evidence from human and animal studies suggests that maternal obesity programs the offspring for increased risk of developing obesity, hyperglycemia, diabetes, hypertension and metabolic syndrome [108].

**Figure 2:**
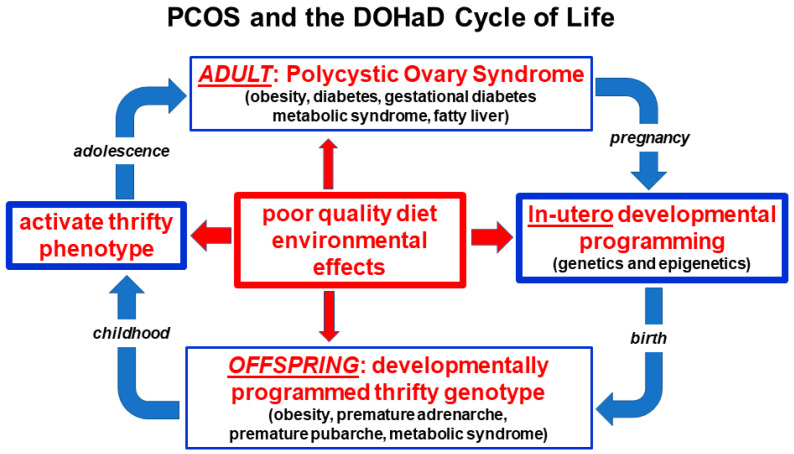
Nutritional and environmental influences throughout the life course and the perpetuation of the transgenerational inheritance of polycystic ovary syndrome. Reprinted from Ref. [28].

The developmental origins of PCOS may have been due to different factors in ancestral and modern populations [17,60]. It has been hypothesized that environmental stress, infection, nutrient deprivation, fetal growth restriction and stress hormone responses may have resulted in maternally mediated modulation of gene expression in ancestral offspring [17,110]. Some of these factors have been investigated and confirmed in modern populations subject to starvation and extreme environmental conditions [111]. In contrast, altered fetal programming in modern societies may be secondary to maternal overnutrition, sedentary behavior, obesity, emotional stress, circadian rhythm disruption, poor gut health or environmental chemical exposure [35,101,112,113]. The preconception and pregnancy periods therefore provide a unique opportunity for lifestyle interventions that promote optimal future health for both the mother and the offspring (Figure 2).

### 3.4. Microbiome and Dysbiosis

The gastrointestinal microbiome is now appreciated to play a central role in human health and disease [114,115]. The microbiome is known to co-regulate many physiological functions involving the immune, neuroendocrine and metabolic systems via complex reciprocal feedback mechanisms that operate between the microbial ecosystem and the host [116,117]. Evidence from studies in Western populations, hunter–gatherer societies and phylogenetic studies in other species, have attempted to place the human microbiome into an evolutionary context [118]. Although microbes clearly impact host physiology and have changed along branches of the evolutionary tree, there is ongoing debate regarding whether the microbiome can evolve according to the usual evolutionary forces [119,120]. Nevertheless, it has been argued that focusing on functional pathways and metabolic roles of microbial communities, rather than on specific microbes, provides a better model for understanding evolutionary fitness [118]. The co-evolution of the microbiome and human physiology may therefore be important in understanding the differences between ancient adaptive physiological survival mechanisms and modern lifestyle-related pathological responses, in women with PCOS (Figure 1).

Twin studies and GWAS show that host genetics can influence the microbiome composition, and microbes can exert effects on the host genome, although the environment has an important role [121,122]. Humans are constantly adapting to the gut microbiome to try to determine which microorganisms are beneficial or harmful. Immune genes involved in this process are the most rapidly evolving protein-encoding genes in the mammalian genome [123,124]. Diversification of microbes allows humans to access dietary niches and nutritional components they otherwise would not be able to access, which may be beneficial and ultimately lead to the integration of specific microbes into the ecosystem [125]. Although no living population today carries an ancestral microbiome, comparison studies of non-Western and Western populations show significant differences in the relative abundances of common phyla and a much greater species diversity in non-Western populations [126,127]. A review of non-human primate and human gut microbiome datasets, revealed a changing microbiome in response to host habitat, season and diet, although there appear to be common species-specific symbiotic communities [118].

Rapid human cultural changes have resulted in significant dietary modifications in urban-industrialized communities and shifted the microbiome at an unprecedented rate. The result has been the development of a mismatch between human metabolic genes and bacteria that enhance fat storage [128]. In our evolutionary past, when nutrients were scarce, it has been theorized that host selection led to the maintenance of microbes that enhance nutrient uptake or host energy storage. However, in the modern environment, where a high-fat, high-sugar, low-fiber diet has become common and easily accessible, integration of these microbes leads to maladaptive physiological responses [40]. For metabolically thrifty individuals with PCOS, harboring microbes that enhance energy storage escalates the evolutionary conflict, furthering the development of insulin resistance and therefore progression to obesity and type 2 diabetes [12,129]. Further compounding this maladaptive response is the loss of microbes that are required to access other dietary niches. One example is the loss of symbiotic species of Treponema in individuals living in urban-industrialized communities [130]. A change from the ancestral hunter–gatherer diet, where foods consumed changed seasonally and a wide variety of food components were eaten, to a diet that is similar across seasons and significantly less varied, is another likely contributor to reduced diversity of the microbiomes of individuals living in urbanized–industrialized communities [131].

The majority of women with PCOS are overweight or obese and evidence indicates that the microbiome of obese individuals is capable of extracting more energy from the host diet compared with the microbiome of lean individuals [132]. This is thought to be driven by an expansion in pro-inflammatory species of bacteria, such as *E. coli*, and a depletion of anti-inflammatory bacteria such as *Faecalibacterium prausnitzii* [133,134]. Chronic low-grade ‘metabolic’ inflammation, or meta-inflammation, is a result of an imbalanced gut microbiome that promotes the development of insulin resistance and type 2 diabetes [135,136,137].

The dysbiosis of gut microbiota theory of PCOS, proposed by Tremellen in 2012, accounts for the development of all of the components of PCOS (multiple ovarian follicles, anovulation or menstrual irregularity and hyperandrogenism) [40]. The theory proposes that a poor-quality diet and resulting imbalanced microbiome, induces intestinal permeability and endotoxemia, exacerbating hyperinsulinemia. Increased insulin levels promote higher androgen production by the ovaries and disrupts normal follicle development. Metabolic, endocrine and environmental factors associated with PCOS are not mutually exclusive, and therefore their relative contributions to dysbiosis in PCOS remains uncertain [138]. Consuming a balanced diet that is low in fat and high in fiber, can also restore balance to the ecosystem (termed eubiosis) [50]. A recent study showed that dietary intake of fiber and vitamin D was significantly decreased in both lean and obese women with PCOS, compared to healthy controls, and correlated with lower diversity of the gut microbiome [139]. Dysbiosis is reversible with improvement in diet quality augmented by the addition of probiotics or synbiotics [51,56,57,58].

Dysbiosis is a consistent finding when looking at the microbiome of women with PCOS [140,141,142,143]. Although most studies are small, dysbiosis has consistently been found to correlate with different physiological parameters, such as obesity, sex hormones and metabolic defects [140,141,143]. Similar to microbiomes associated with obesity, the microbiomes of individuals with PCOS have generally been found to have lower alpha diversity (lower numbers of bacterial taxa) than controls, and most studies describe an altered composition of taxa relative to controls [140,143]. However, the bacterial taxa observed to be either increased, depleted or absent in PCOS differs from study to study. This is likely due to both the immense inter-individual variation in microbiotas, as well the fact that PCOS is a quantitative trait with women with various degrees and levels of obesity and sex hormones.

In keeping with the developmental origins hypothesis previously discussed, maternal androgens may alter the composition and function of the microbiome, therefore facilitating the pathogenesis of PCOS [140]. One study showed that beta diversity, which is used to measure differences between groups, was negatively correlated with hyperandrogenism, suggesting that androgens play a significant role in dysbiosis [140]. The ‘first hit’ in utero may therefore combine with vertical transmission of a dysbiotic microbiome from a mother with PCOS, resulting in dysbiosis in the offspring. Preconception and pregnancy provide a unique opportunities for lifestyle and dietary interventions aimed at restoring eubiosis, to enable the transference of a balanced ecosystem to the offspring, via vertical transmission [118].

The accumulating scientific evidence strongly supports the significant role played by the microbiome in the pathogenesis and maintenance of PCOS, consistent with research in other related metabolic conditions. The role of dysbiosis is supported by over 30 proof-of-concept studies that have recently been reviewed [144]. Dysbiosis is therefore a significant factor in the pathogenesis of PCOS and an important component of a unified evolutionary model. Dysbiosis represents a maladaptive response of the microbiome to modern lifestyle influences and is a modifiable factor in the treatment of women with PCOS.

### 3.5. Insulin Resistance

There are several dilemmas when assessing the role of IR in women with PCOS. There is no consensus on the definition of IR [145,146], measurement is difficult [147,148], whole-body IR is usually measured although it is recognized that IR can be selective being either tissue-specific or pathway-specific within cells [149,150,151], normal values are categorical and determined by arbitrary cut-offs (4.45 mg/kg/min) [145], testing is not recommended in clinical practice [38], reported prevalence rates in obese and lean women vary widely [147,152], and the significance of IR as a pathognomonic component of PCOS is an area of debate [153,154,155].

Despite these limitations, it is hypothesized that IR is a significant proximate cause of PCOS and is intrinsic to the underlying pathophysiology [44,156]. In addition, it is recognized that IR plays a major role in the pathophysiology of all of the metabolic diseases, cardiovascular disease, some neurodegenerative diseases, and selected cancers [22,157]. Insulin resistance is therefore considered to be the main driver for many diseases and makes a significant contribution to the chronic disease epidemic [158]. Nevertheless, being able to vary the sensitivity and physiological action of insulin is thought to have conferred a significant adaptive survival role in many animals throughout evolutionary history [146,159]. It has been proposed that IR may have evolved as a switch in reproductive and metabolic strategies, since the development of IR can result in anovulation and reduced fertility, in addition to differential energy repartitioning to specific tissues [159].

Insulin receptors are located on the cell membranes of most tissues in the body [160]. Ligand binding to the alpha-subunit induces autophosphorylation of specific tyrosine residues on the cytoplasmic side of the membrane [160,161]. The activated insulin receptor initiates signal transduction via the phosphatidylinositol-3 kinase (PI-3K) metabolic pathway and the mitogen-activated protein kinase pathway (MAPK) which is involved in cell growth and proliferation [161]. Insulin is an anabolic hormone that facilitates glucose removal from the blood, enhances fat storage and inhibits lipolysis in adipose tissue, stimulates glycogen synthesis in muscle and liver and inhibits hepatic glucose output [161]. IR can be defined as a state where higher circulating insulin levels are necessary to achieve an integrated glucose-lowering response [146]. IR results from alterations to cellular membrane insulin-receptor function or intracellular signaling, enzyme, metabolic or gene function [146,160,161].

Insulin resistance can be caused by a wide variety of mechanisms that have the ability to disrupt any part of this metabolic signaling system [53,161]. These include autoantibodies, receptor agonists and antagonists, hormones, inflammatory cytokines, oxidative stress, nutrient sensors and metabolic intermediates [160,161,162,163]. Physiological regulation of insulin function can be viewed as an adaptive mechanism to regulate the metabolic pathway of insulin signaling (PI-3K), in response to changing environmental conditions [starvation, fear, stress] [164,165] or during normal alterations of internal states (pregnancy, lactation, adolescence) [65,146,152].

The physiological activation of IR allows the organism to switch from an anabolic energy storage state to a catabolic or energy mobilizing state. This allows free fatty acids to be mobilized from adipose tissue, which are then converted to glucose in the liver and released into the circulation [161]. As a result of this metabolic change, blood sugar levels are maintained for vital metabolic processes and brain function [14]. This adaptive protective mechanism can be pathway-specific during periods of growth, such as pregnancy, lactation and adolescence, so that only the metabolic signaling (PI-3K) is inhibited and not the mitogenic pathway (MAPK), which may even be up-regulated [30,65,160].

When the physiology of insulin function is considered to be a quantitative or continuous variable from an evolutionary perspective, it is likely that all women with PCOS, whether obese or lean, have reduced insulin sensitivity [152,155,166]. A systematic review and meta-analysis of euglycemic-hyperinsulinemic clamp studies found that women with PCOS have a 27% reduction in insulin sensitivity compared to body mass index (BMI) and age-matched controls [155]. In evolutionary terms, women with a PCOS metabolic phenotype would have increased survival chances during times of environmental or physiological demand for altered energy metabolism, but be more vulnerable to the pathological effects of IR when exposed to modern lifestyle factors [14,17,159]. In particular, a poor-quality, high-glycemic, high-fat, low-fiber diet has been shown to cause IR [40,167]. As discussed in the dysbiosis section, diet-related changes in the gastrointestinal microbiome have also been shown to cause IR in women with PCOS [53,55]. Numerous studies have shown that dietary modification [168,169,170], or treatment with probiotics or synbiotics, has the potential to restore normal insulin function [57,171].

Consumption of a high-glycemic-load diet results in rapid increases in blood sugar levels that cause compensatory hyperinsulinemia [167,172]. Excessive dietary intake of glucose and fructose are converted to fatty acids by de novo lipogenesis in the liver, transported to adipocytes via lipoproteins, released as fatty acids to adipocytes and stored in fat globules as triglycerides [161]. As a result of nutrient overload, diacylglycerol, the penultimate molecule in the synthesis of triglyceride, accumulates in the cytoplasm and binds with the threonine amino acid in the 1160 position of the insulin receptor. This inhibits autophosphorylation and down-regulates the metabolic PI-3K pathway and causes IR [161]. This process has the potential to be reversible following changes in diet quantity and quality, as has been shown to occur with calorie restriction, fasting, time-restricted eating, gastric bypass surgery, low saturated fat and low glycemic diets [168,170,173]. Diets high in animal protein or saturated fat can also cause IR independent of BMI [174,175]. These mechanisms provide the rationale for the principal recommendation of the International Guidelines that women with PCOS should be advised about dietary modification as the first line of management in all symptom presentations [38].

### 3.6. Obesity and the Lean PCOS Paradox

Insight can be obtained into the role of obesity in women with PCOS by examining the evolutionary history, genetic studies and pathological disorders of adipose tissue [151,176,177]. The ability to store energy is a basic function of life beginning with unicellular organisms [176]. In multicellular organisms, from yeast to humans, the largest source of stored energy is as triglycerides in lipid droplets in order to provide energy during periods when energy demands exceed caloric intake [176]. Understanding the biological functions of adipose tissue has progressed from energy storage and thermal insulation to that of a complex endocrine organ with immune and inflammatory effects and important reproductive and metabolic implications [176,178].

Adipose tissue is organized into brown adipose tissue (BAT) and white adipose tissue (WAT), both with different functions [178]. Although the evolutionary origins of BAT and WAT are the subject of ongoing debate [176], BAT is located in the supraclavicular and thoracic prevertebral areas and is primarily involved in cold thermogenesis and regulation of basal metabolic rate [179]. WAT is distributed in multiple anatomical areas such as visceral adipose tissue (VAT) and subcutaneous adipose tissue (SAT) and functions as a fat storage depot and an endocrine organ [178,179]. An additional layer of SAT is thought to have evolved as insulation against cool night temperatures in the Pleistocene open Savanah [180]. The lower body distribution of SAT in women is hypothesized to have evolved to provide additional calorie storage for pregnancy and lactation and is unique to human females [14]. Lower body SAT has a metabolic program that makes it less readily available for every-day energy needs, but it can be mobilized during pregnancy and lactation [14]. In addition, excess accumulation of SAT is much less likely to cause IR and metabolic dysfunction and explains why IR is not observed in all obese individuals [151,181]. Visceral WAT is associated with IR in women with PCOS leading to both metabolic and reproductive problems [182].

Multiple lines of evidence from evolutionary history, genetic and twin studies, support a genetic basis for obesity and differences in obese and lean phenotypes in women with PCOS [183,184,185,186]. The majority of women with PCOS are overweight or obese, with reports ranging from 38–88% [152,186]. Studies comparing obese and lean women with PCOS have several methodological problems including small sample size, overlap of PCOS characteristics with normal pubertal changes, non-standardized diagnostic criteria, and limited generalizability to the entire population due to a focus on a specific ethnic group [166,182]. In addition, most of the studies examining body composition in PCOS have relied on anthropomorphic measurements (BMI, waist circumference, waist-to-hip ratio) which are considered inaccurate compared with the current gold-standard of magnetic resonance imaging [182]. Consequently, there is wide heterogeneity in reports examining the relationship between body composition measures, including extent of VAT and metabolic changes such as IR [186].

In humans, there is large individual variation in the fat storage capability and expandability of different adipose tissue depots [151]. It has been hypothesized that once the genetically determined limit of expandability of SAT is reached, there is expansion of VAT and excess lipid accumulation in muscle, liver and other organs, resulting in IR, inflammation and metabolic dysregulation [151]. We hypothesize that lean women with PCOS have a genetically determined limited ability to store excess lipid in SAT, but develop increased lipid deposition in VAT and organs such as the liver, resulting in metabolic dysregulation and IR in a similar manner to what occurs in obese women with PCOS. The wide variation in the genetic limitation of SAT expansion is also supported by studies in individuals with lipodystrophy.

Lipodystrophies are a heterogenous group of rare inherited and acquired disorders characterized by a selective loss of adipose tissue [177,187]. They are classified on the basis of the extent of fat loss as generalized, partial or localized [187]. Patients with congenital generalized lipodystrophy have a generalized deficiency of fat from birth, usually have severe IR and develop diabetes at puberty. As a consequence of genetically limited ability for SAT lipid storage, lipids can only be stored ectopically in non-adipocytes resulting in major health consequences including IR, fatty liver, diabetes and PCOS [188]. In contrast to generalized lipodystrophy, patients with familial partial lipodystrophy have normal fat distribution at birth but loose SAT in the limbs, buttocks and hips, at puberty. Fifty percent of women develop diabetes and 20–35% develop irregular periods and polycystic ovaries [177]. Despite the rare nature of these syndromes much has been learned about the underlying genetic variants involved [187].

Elucidation of clinical subtypes and the genetic background of patients with lipodystrophies may pave the way to new insights into the role of fat partitioning and obesity, and has implications for understanding the pathogenesis of insulin resistance, diabetes and PCOS [177]. Lean women with PCOS may have a genetic predisposition for limited SAT fat storage, coupled with underlying metabolic predispositions that result in deposition of excess lipid in VAT and liver and the observed metabolic features of IR, fatty liver and diabetes. If the extent of IR and ectopic fat deposition is excessive, the resulting hormonal changes may be sufficient to cause oligomenorrhoea and subfertility as occurs with secondary familial partial lipodystrophy type 2 [188,189]. If this underlying mechanism is confirmed in future studies, the main difference between women with lean or obese PCOS may be the combined effects of metabolic programming and the genetically determined extent of SCT fat deposition. This would explain why lean women have all the same clinical, biochemical and endocrine features, although possibly less severe, than overweight and obese women with PCOS [186].

### 3.7. Endocrine-Disrupting Chemical Exposure

Anthropomorphic chemical exposure is ubiquitous in the environment and has possible effects on many aspects related to women’s health and PCOS [36,190,191,192]. The identification of more than 1000 EDC in food, air, water, pesticides, plastics, personal care products, and other consumer goods, raises specific concerns for pregnant women and women with increased susceptibility to metabolic diseases such as PCOS [36,172,192,193,194]. Accumulating evidence suggests that EDC may be involved in the pathogenesis of PCOS given their known and potential hormonal and metabolic effects [36,190,195]. This includes many of the areas that have been considered in the unified evolutionary model, such as developmental epigenetic programming, microbiome composition and function, metabolic processes such IR, and regulation of body weight.

Many observational studies have demonstrated the presence of EDC in maternal and fetal serum and urine, amniotic fluid, cord blood and breast milk [196,197,198]. Six classes of EDC have been shown to cross the placenta confirming that the fetus is exposed at all stages of development [109,196]. Although it is impossible to perform experimental studies in humans, evidence from epidemiological, molecular toxicology and animal studies provide compelling evidence of adverse developmental effects and transgenerational toxicity [172,190,192,199]. The realization of the tragic effects of DES in the 1970′s was first example of an in utero exposure causing serious transgenerational health effects [192].

Several estrogenic EDC have been associated with birth outcomes that are thought to be associated with the development of PCOS [190]. These include decreased birthweight (perfluoroakyl substances [PFAS], perfluorooctanoic acid) and preterm birth (di-2-ethylhexyl phthalate) [190]. Prenatal exposure to androgenic EDC (triclosan, glyphosate, tributyltin, nicotine) is of increasing concern, given the suspected epigenetic role of in utero androgen exposure in the pathogenesis of PCOS [48,200,201].

As a result, implementation of the precautionary principle is a high priority in counselling women with PCOS [202]. International professional bodies (The Royal College of Obstetricians and Gynecologists, Endocrine Society, FIGO) have recommended that all pregnant women should be advised of the possible risks of EDC and that education programs be developed to inform health professionals [203,204,205]. An explanation of the pathogenesis of PCOS should include reference to environmental chemical exposure and open the way for more detailed discussion of specific personalized advice and lifestyle recommendations.

### 3.8. Lifestyle Contributors to the Pathogenesis of PCOS

Several lifestyle factors have been investigated for their role in the pathogenesis of PCOS. These include diet, exercise, stress, sleep disturbance, circadian disruption and exposure to environmental chemicals [28,41,206]. Recent advances in genomics, epigenetics, metabolomics, nutrigenomics, evolutionary biology, computer technology and artificial intelligence, are providing many insights into the mechanisms of how lifestyle factors impact the pathogenesis of PCOS [9,90,207,208]. Nutritional studies based on diet indices, diet composition and metabolomics have identified dietary components that contribute to a healthy eating pattern [51,207,209,210]. Healthy diet patterns, or wholefood diets, have been found to be effective in controlling and reversing many of the symptoms and metabolic alterations associated with PCOS [50].

As previously discussed, the modern Western diet and lifestyle is at odds with our evolutionary background. One dietary component that differs significantly in ancestral and modern populations is dietary fiber intake. Assessment of dietary fiber intake is also a good surrogate marker for a healthy wholefood diet. In general, our traditional hunter–gatherer ancestors consumed significantly more fiber than modern populations. Studies that have investigated the dietary patterns of remaining contemporary hunter–gatherer societies, have found their dietary fiber intake to be around 80–150 g per day [211]. This contrasts with the contemporary Western diet, where the average fiber intake is 18.2 g per day in children and 20.7 g per day in adults [212]. Adequate dietary fiber consumption is important as it has several benefits, such as improved insulin sensitivity, reduced blood glucose levels, decreased systemic inflammation, lower serum levels of androgens and LPS, all of which have been linked to the pathogenesis of PCOS [213,214,215,216].

Recent systematic reviews of observational studies and randomized controlled trials have found dietary fiber consumption to be inversely related to risk of obesity, type 2 diabetes, and cardiovascular disease [217,218]. A recent cohort study from Canada found that obese women with PCOS consumed significantly less dietary fiber than normal weight women without PCOS [219]. In addition, fiber intake of women with PCOS was negatively correlated with IR, fasting insulin, glucose tolerance and serum androgens [219]. Hence, the mismatch between the amount of fiber traditionally consumed and the fiber content of Western diets, may be an important dietary component contributing to the increased rates of PCOS seen in developed and developing nations.

### 3.9. Circadian Rhythm Disruption and PCOS

The circadian rhythm is a mechanism with which living organisms can synchronize their internal biological processes with the external light and dark pattern of the day [220]. Circadian rhythms have formed a central component of the evolutionary adaptation of all organisms to a variety of environmental conditions, from procaryotes to complex multicellular organisms [221,222,223]. Most organisms experience daily changes in their environment, including light availability, temperature and food. Hundreds of thousands of years of evolution have synchronized the rhythmic daily programming of internal metabolic, endocrine and behavioral systems to the external environmental conditions [222]. Circadian clocks anticipate environmental changes and confer a predictive adaptive survival benefit to organisms.

The normal function of the circadian system is based on a hierarchical network of central and peripheral clocks [224]. The central, or master clock, is in the suprachiasmic nucleus in the anterior hypothalamus. It is strategically placed to communicate with multiple physiological homeostatic control nuclei (body temperature, metabolic rate, appetite, sleep), pituitary hormonal systems (gonadal, thyroid, somatotrophic, adrenal), the autonomic nervous system (digestion, heart rate), and conscious cortical centers (behavior, motivation, reward, reproduction) [225]. Humans are programmed for specific day and night-time survival behaviors that are regulated by the availability of temperature, feeding and sunlight. Photons of light stimulate specialized photoreceptors in the retinal ganglion layer which transmit an electrical impulse to the cells of the master clock via the retinohypothalamic tract [226]. The central clock can then convey rhythmic information to peripheral clocks in other tissues and organs throughout the body [224]. Feeding and fasting cycles are the primary time cues for circadian clocks in peripheral tissues [227].

Circadian clocks exist in all cells, including the microbiome, and function as autonomous transcriptional-translational genetic feedback loops [228,229]. The changing length of daylight, determined by the rotation of the earth on its axis, requires that the autonomous clocks are reset, or entrained, on a daily basis [230]. The molecular mechanisms of circadian clocks are similar across all species and are regulated by genetic enhancer/repressor elements, epigenetic modulation by methylation and acetylation, post-translation modification of regulatory proteins, and a variety of hormonal and signaling molecules [220,229,231]. This complex interconnected regulatory framework, ensures that the same molecules that regulate metabolism and reproduction, also contribute to a bidirectional feedback system with the autonomous circadian circuits [224,231]. This results in synchronicity of internal physiology with environmental cues, to optimize both individual and species survival. Evolution has therefore provided a mechanism for humans to adapt and survive under the selective pressures of food scarcity, seasonal changes in sunlight and a range of temperature exposures.

The evolutionary adaptive survival benefit of synchronized circadian systems in ancient populations is in marked contrast to the multiple circadian disruptions that are associated with modern lifestyle. These include poor-quality diet [232], improper meal timing and altered feeding-fasting behavior [233,234], sub-optimal exercise timing [235], disrupted sleep-wake cycles [236], shift work [237], EDC [238], and stress [239,240]. Changes in all of these parameters are correlated with significant increases in obesity, diabetes, cardiovascular disease, and some cancers [222]. Not surprisingly, lifestyle-related disturbances of circadian rhythms have also been investigated for their role in the pathogenesis of PCOS [35,241,242]. The available evidence suggests that circadian disruption has detrimental effects on in utero development [243], altered metabolism and insulin resistance [241,244], body weight and obesity [245], and fertility [34]. All these influences are relevant to an evolutionary model of the pathogenesis of PCOS.

Recognition of the impact of lifestyle behaviors on circadian dysregulation and metabolic and reproductive function, opens the way for targeted intervention strategies to modulate and reverse these effects [246]. These include regular meal timing [222,247], time-restricted feeding [248,249], restoration of normal sleep cycles [250], optimal exercise timing [235], limitation of exposure to bright light at night [251], and improved diet quality [227]. Recognition of circadian dysfunction and the investigation of lifestyle interventions should be a priority in both clinical management and future research in PCOS.

### 3.10. Conceptual Framework and Summary of the Unified Evolutionary Model

The evolutionary model proposes that PCOS is a condition that arises from the inheritance of genomic variants derived from the maternal and paternal genome. In utero fetal metabolic, endocrine and environmental factors modulate developmental programming of susceptible genes and predispose the offspring to develop PCOS. Postnatal exposure to poor-quality diet, sedentary behavior, EDC, circadian disruption and other lifestyle factors activate epigenetically programmed pathways, resulting in the observed features.

Dietary factors cause gastrointestinal dysbiosis and systemic inflammation, insulin resistance and hyperandrogenism. Continued exposure to adverse lifestyle and environmental factors eventually leads to the development of associated metabolic conditions such as obesity, GDM, diabetes, NAFLD and metabolic syndrome (Figure 1).

Balanced evolutionary selection pressures result in transgenerational transmission of susceptible gene variants to PCOS offspring. Ongoing exposure to adverse nutritional and environmental factors activate developmentally programmed genes and ensure the perpetuation of the syndrome in subsequent generations. The DOHaD cycle can be interrupted at any point from pregnancy to birth, childhood, adolescence or adulthood by targeted intervention strategies (Figure 2).

In summary, we propose that PCOS is an environmental mismatch disorder that manifests after in utero developmental programming of a cluster of normal gene variants. Postnatal exposure to adverse lifestyle and environmental conditions results in the observed metabolic and endocrine features. PCOS therefore represents a maladaptive response of ancient genetic survival mechanisms to modern lifestyle practices.

**Figure 3:**
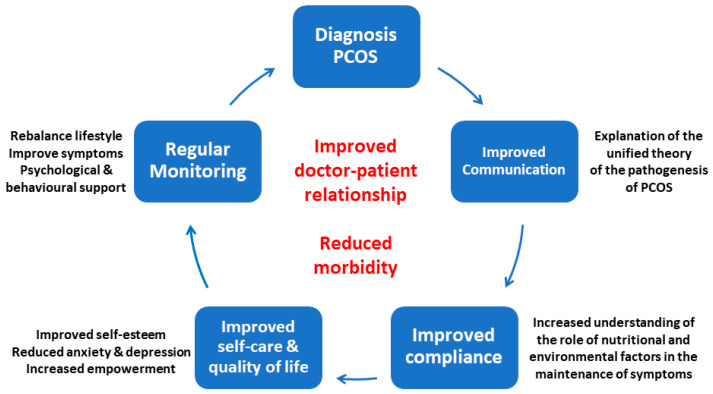
Impact of the unified theory on the management of polycystic ovary syndrome. Reprinted from Ref. [28].

Comprehensive International Guidelines have made 166 recommendations for the assessment and management of PCOS [38]. We believe the current unified evolutionary theory of the pathogenesis of PCOS provides a conceptual framework that may help practitioners and patients understand the development of PCOS symptoms and pathology in the context of our modern lifestyle and environment. It will hopefully contribute to improved communication, result in improved feelings of empowerment over the personal manifestations of PCOS, improve compliance, reduce morbidity, increase quality of life and inform future research (Figure 3).

## 4. Conclusions

Substantial evidence and discussion support an evolutionary basis for the pathogenesis of polycystic ovary syndrome, although many of the mechanistic details are yet to be determined. Nevertheless, multiple lines of evidence from evolutionary theory, comparative biology, genetics, epigenetics, metabolism research, and cell biology, provide supportive evidence and hypothesis-generating data. The ability of animals to synchronize internal physiology, metabolism and reproductive function, with our changing external environment and habitat, are a necessary requirement for individual and species survival. The co-operative and sometimes competitive evolution of metabolism and reproduction provided adaptive survival mechanisms in ancestral environments that appear to be maladaptive in modern environments. An evolutionary model therefore provides a framework to enhance practitioner and patient understanding, improve compliance with lifestyle interventions, reduce morbidity, improve quality of life and will evolve and change over time.

## Data Availability

Not applicable.
